# Gold Nanoparticles-Coated SU-8 for Sensitive Fluorescence-Based Detections of DNA

**DOI:** 10.3390/diagnostics2040072

**Published:** 2012-11-29

**Authors:** Cuong Cao, Sam W. Birtwell, Jonas Høgberg, Hywel Morgan, Anders Wolff, Dang Duong Bang

**Affiliations:** 1 DTU-NANOTECH, Department of Micro and Nanotechnology, Technical University of Denmark, DTU Building 423, DK-2800 Kongens Lyngby, Denmark; Email: ccao@ntu.edu.sg (C.C.); anders.wolff@nanotech.dtu.dk (A.W.); 2 School of Electronics and Computer Science, University of Southampton, Southampton SO17 1BJ, UK; Email: s.birtwell@soton.ac.uk (S.W.B.); hm@ecs.soton.ac.uk (H.M.); 3 DTU-VET, Diagnostic Laboratory, National Veterinary Institute, Technical University of Denmark, Bülowsvej 27, DK-1870 Frederiksberg C, Denmark; Email: jonho@vet.dtu.dk; 4 DTU-FOOD, Laboratory of Applied Micro-Nanotechnology (LAMINATE), Diagnostic Engineering, Division of Food Microbiology, National Food Institute, Technical University of Denmark, Mørkhøj Bygade 19, DK-2860 Søborg, Denmark

**Keywords:** gold nanoparticles, SU-8, autofluorescence, solid-phase PCR amplification, DNA hybridization

## Abstract

SU-8 epoxy-based negative photoresist has been extensively employed as a structural material for fabrication of numerous biological microelectro-mechanical systems (Bio-MEMS) or lab-on-a-chip (LOC) devices. However, SU-8 has a high autofluorescence level that limits sensitivity of microdevices that use fluorescence as the predominant detection workhorse. Here, we show that deposition of a thin gold nanoparticles layer onto the SU-8 surface significantly reduces the autofluorescence of the coated SU-8 surface by as much as 81% compared to bare SU-8. Furthermore, DNA probes can easily be immobilized on the Au surface with high thermal stability. These improvements enabled sensitive DNA detection by simple DNA hybridization down to 1 nM (a two orders of magnitude improvement) or by solid-phase PCR with sub-picomolar sensitivity. The approach is simple and easy to perform, making it suitable for various Bio-MEMs and LOC devices that use SU-8 as a structural material.

## 1. Introduction

The epoxy-based negative photoresist SU-8 has been extensively used as a structural material for fabrication of numerous Bio-MEMS or LOC devices owing to its ease of fabrication, thermal and chemical stability, and optical transparency [1]. Though useful, the SU-8 also has a major disadvantageous attribute, *i.e.*, the SU-8 possesses complex ring structures that may act as a source of high autofluorescence at visible wavelengths [2]. Its autofluorescence level is high compared to other photo-patternable polymers, such as polydimethylsiloxane, silicone, cyclic olefin copolymer, polystyrene, *etc.* [3]. Many Bio-MEMS or LOC devices rely on fluorescence detection, and the high background fluorescence generated by SU-8 therefore limits its use as a substrate. However, only a few studies have focused on the reduction of SU-8 autofluorescence in order to realize its full potential for applications in biomedical diagnostics [2]. 

Attempts to develop a highly functionalized SU-8 surface with a high immobilization efficiency and good accessibility to target biomolecules have also been the major concerns [4,5]. Nucleic acids, antigens or antibodies can be immobilized on SU-8 by physical adsorption, but this method is not favorable in terms of stability and accessibility of the biomolecules. Covalent immobilization of biomolecules on SU-8 usually requires multiple steps: the SU-8 should be modified to have at least one desired functional group, *i.e*., amine, thiol, aldehyde, carboxyl, *etc.*, for covalent binding of biomolecules [6]. Such methods will result in better biofunctionality, less non-specific adsorption and higher stability. However, these methods usually use strong chemicals (acids or bases) or plasma treatments, which may damage the adjacent micro- and nanostructures of the LOC devices physically while modifying the area of interest [4,7]. 

Matrices consisting of both polymers and inorganic materials offer great benefits. Coupled with the ease of polymer microfabrication processes, the inorganic phase, *i.e*., planar gold film or gold nanoparticles (AuNPs), can provide LOC devices with new functionalities regarding conductivity [8], optics [9], catalysis [10] or near-field surface effects [11]. Here, we show that deposition of a thin AuNPs layer onto a SU-8 surface significantly reduces the autofluorescence of the coated SU-8 surface to much less than that of bare SU-8. Furthermore, DNA probes can easily be immobilized onto the Au surface, and these are thermally stable over a wide range of temperatures, thus providing high-performance SU-8-based biosensors for, *e.g*., DNA hybridization and solid-phase PCR (SP-PCR).

## 2. Experimental Section

### 2.1. Chemicals and Materials

Ethanolamine, sodium phosphate buffer (pH 7), isopropyl alcohol, 3-aminopropyltriethoxy silane (APTES), hydrogen tetrachloroaurate trihydrate, sodium citrate and PerfectHyb^TM^ Plus hybridization buffer were obtained from Sigma-Aldrich A/S, Denmark. All other essential chemicals were of analytical grade unless otherwise stated. Chemical solutions and buffers were prepared in ultra-pure water (18.2 MΩ/cm) when required. All primers and probes were purchased from TAG Copenhagen A/S, Denmark. 

### 2.2. Preparation of the SU-8 Microparticles

The SU-8 material used in this study is SU-8 barcode microparticles that were fabricated by single-step photolithography in SU-8 photopolymer, a common novalac-epoxy based photoresist used in many micro-device applications [12,13]. Briefly, cleaned <100> p-type silicon wafers were coated with Ti-prime adhesion coater (MicroChemicals GmbH, Ulm, Germany), followed by spinning SU-8-25 (Chestech Ltd., Rugby, UK) at 2,000 rpm to give a coating of 26 ± 1 µm. The SU-8 was soft-baked for 3 min at 65 °C, followed by ramping to 95 °C at 10 °C/min and held at 95 °C for 8.5 min. The wafer was rested for 20 min to alleviate stress and exposed through a mask to 104 mJ/cm^2^ UV radiation (wavelength 360 nm) in an EVG 620 mask aligner. A post exposure bake (PEB) was then performed for 2 min at 65 °C, followed by ramping to 95 °C at 10 °C/min and held at 95 °C for 4 min. Particles were rested for 12 h to reduce wafer stress and prevent cracking during development. Finally, the particles were developed for 4 min 20 s in Rohm and Haas EC solvent (Chestech Ltd., Rugby, UK). Each wafer contained 8,000 particles in eight square blocks of 1,000. Each block of particles on the wafer is identified by a different unique code. 

After manufacture, the particles were removed from the wafer by etching the silicon using a 2% tetramethylammonium hydroxide solution in water with 0.02% surfactant (Rohm and Hass MF319, Chestech Ltd., Rugby, UK) at 80 °C for 30 min. The particles were washed four times in water with 0.02% v/v Tween-20^®^ surfactant (Sigma-Aldrich, Poole, UK) and resuspended in the same solution, ready for use. 

### 2.3. Surface Modification and Functionalization of the SU-8 Microparticles (Scheme 1)

The SU-8 microparticles are first hydroxylated by incubating with 0.1 M ethanolamine/0.5 M sodium phosphate buffer (pH 7) in an Eppendorf tube for 10 hr with gentle mixing (Step 1). Then, the hydroxylated SU-8 microparticles are washed with isopropyl alcohol. A 2% (v/v) solution of 3-aminopropyltriethoxy silane (APTES) in isopropyl alcohol is added to the SU-8 microparticles for silanization reaction (Step 2). APTES will provide multiple amine functional groups (three amine groups per APTES molecule) extending outwards as the new termination for AuNPs binding. Therefore, one can maximize the coating of AuNPs, as well as the binding strength, between AuNPs and SU-8. After 90 min of silanization at room temperature, the amine-functionalized SU-8 microparticles are rinsed thoroughly with ultra pure water (18.2 MΩ/cm) and absolute ethanol. Then, 1 mL of 7 × 10^−9^ M spherical AuNPs with an average diameter of 17.8 ± 1.9 nm, synthesized by citrate reduction method [10], is bound to the functionalized SU-8 surface, owing to charge attraction and coordination effect of amino groups (Step 3) [14,15,16]. This process takes about 2 h to complete, but the reaction can be kept overnight to maximize the deposition of the AuNPs. 10 µM thiolated DNA probe (5*'* Thiol-TT TTT TTT TTA GGA AGG TGT GGA CGA CGT CAA GTC ATC ATG GCC 3*'*, working as a nested probe for the SP-PCR described below) prepared in 500 µL DNA-free water is then immobilized on the Au surface by incubating overnight at room temperature with gentle mixing (Step 4). When the self-assembly process is over, the microparticles are washed with boiling deionized water to eliminate excessive and non-specifically bound DNA probes.

**Scheme 1:**
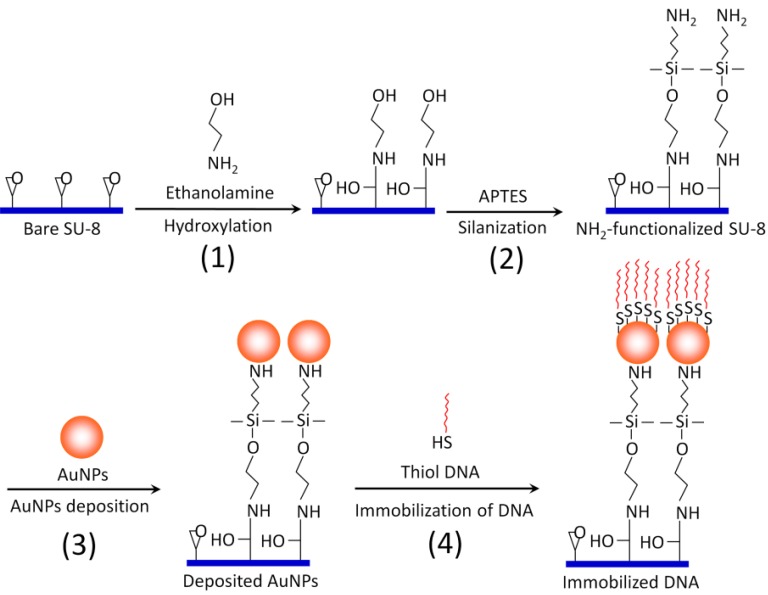
Schematic illustration of overall surface functionalization of SU-8 with AuNPs.

### 2.4. Thermal Stability Investigation of the Immobilized DNA Probes on SU-8 Microparticles

The AuNPs-coated SU-8 microparticles were immerged in 100 µL of water and treated at different temperature courses (room temperature, 95 °C for 20 min and 40 min, and up to 40 repeated 54–94 °C heating/cooling PCR cycles). Then, the SU-8 particles were incubated with various ten-fold dilutions of the probe’s complementary sequence tagged with Cy5 (5*'* GGC CAT GAT GAC TTG ACG TCG TCC ACA CCT TCC T(A)10 Cy5 3*'*) in 0.5× PerfectHyb™ Plus hybridization buffer at 42 °C for 1 h. Control experiments were also performed by hybridizing with a non-complementary sequence labeled with Cy5 (5*'* Cy5 TCG CGR TAT TGC GTC TCA TTG TAT ATG 3*'*, working as a reverse primer for the SP-PCR described below). Finally, the fluorescence signals were measured using a scanner with appropriate settings for the Cy5 fluorophore. 

### 2.5. Solid-Phase PCR (SP-PCR) Amplification

The SP-PCR was performed for the detection of *Campylobacter jejuni (C. jejuni)*, a major food-borne pathogen causing Campylobacteriosis worldwide [17]. The primer set, including a forward primer (5 µM, 5*'* GCG AAG AAC CTT ACC TGG GCT TGA TA 3*'*) and a reverse primer (20 µM, 5*'* Cy5 TCG CGR TAT TGC GTC TCA TTG TAT ATG 3*'*), is used to amplify a 302-bp DNA fragment of the 16S ribosomal gene of *C. jejuni*. The PCR thermocycling includes enzyme activation at 94 °C for 5 min, followed by 35 cycles with denaturation at 94 °C for 15 s, annealing at 54 °C for 20 s and extension at 72 °C for 20 s. The final extension step is carried out at 72 °C for 1 min. The total volume of a PCR mixture was 25 µL, consisting of 12.5 µL PCR Mastermix (Promega Biotech AB, Denmark), 0.1 µL of each primer, 1 µL of 50 mM MgCl_2_, 1 µL of 100 mg/mL BSA, 5.3 µL of DNA-free water, 5 µL of DNA template and the functionalized SU-8 microparticles (~20 particles). The PCR is performed by a PTC-200 Thermal Cycler (Bio-Rad, USA).

### 2.6. Characterizations and Measurements

The SU-8 microparticles were characterized using optical microscopy (Leica DMRB, Leica Microsystems A/S) and a scanning electron microscopy (SEM) equipped with elemental analysis (EDS) (Quanta 200F SEM, operated at 5 kV). Fluorescence measurement was performed by a fluorescence scanner operating in reflection mode (BioAnalyzer 4 EPI, LaVision BioTec GmbH, Germany) with Cy3, Cy5 and FITC filters. 

## 3. Results and Discussion

In this study, the experimental substrate is SU-8 barcode microparticles that have been described elsewhere [6,18], and we used them only as a typical SU-8 material for surface modification and demonstration of implementations, *i.e.*, DNA hybridization and SP-PCR.

**Figure 1:**
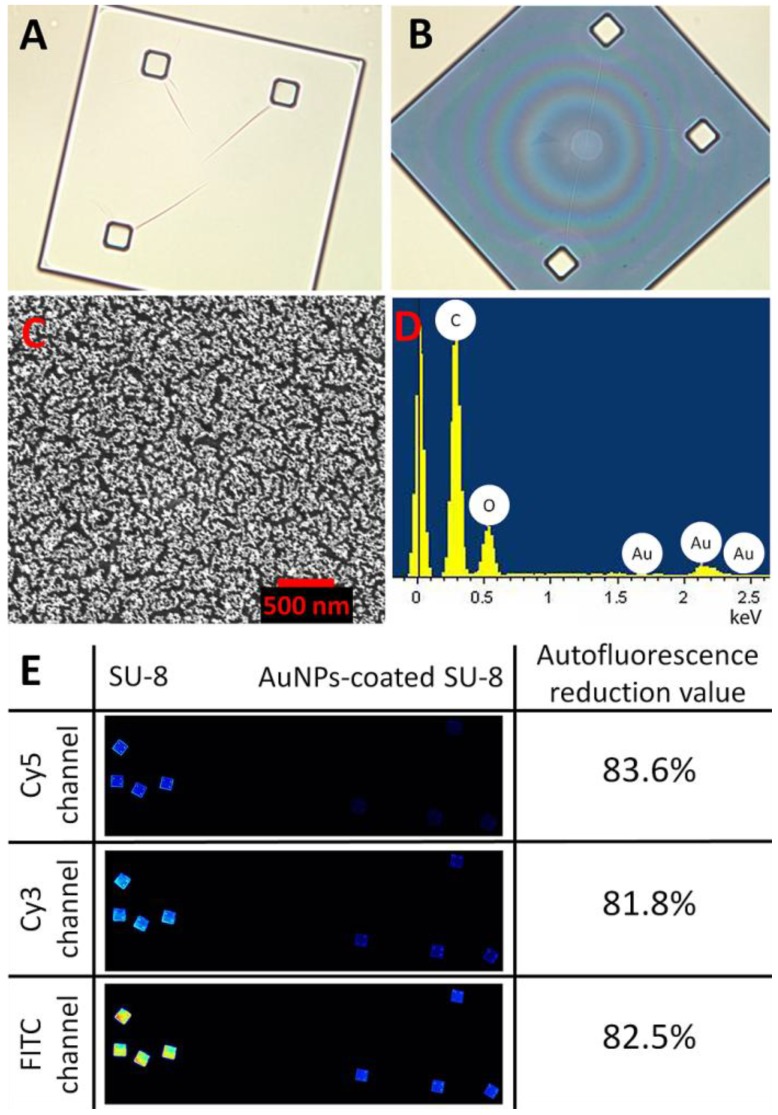
Optical images of a SU-8 microparticle before (**A**) and after (**B**) deposition of the AuNPs (at 200× magnification). (**C**) Surface characterization of a Au-coated SU-8 microparticle by scanning electron microscopy (SEM) and (**D**) energy-dispersive spectroscopy (EDS-SEM) demonstrating the Au layer deposited on the SU-8 surface. (**E**) shows reduction in autofluorescence of the Au-coated SU-8 surface for the different wavelength ranges: Cy3, Cy5 and FITC.

Figure 1 shows optical characterizations of the SU-8 microparticles by optical microscopy, SEM, and EDS. The SU-8 microparticle has a size of 250 × 250 × 5 µm (length x width x thickness) with good transparency at visible wavelengths (Figure 1(A)). After the chemical modification process, the AuNPs were effectively immobilized as a thin monolayer (about 20 nm) on the surface of SU-8 with high homogeneity (Figure 1(C)). The EDS analysis in Figure 1(D) indicates the presence of Au material, confirming that the deposition of the spherical AuNPs was successful. Although the optical transparency (measured as grayscale intensity per pixel using Photoshop CS3 (Adobe Systems Inc., USA)), was reduced from 94% to 63% (Figure 1(A,B)), after the immobilization, the reduction of autofluorescent of the AuNPs-coated SU-8 surface was as much as 81% in comparison to the untreated SU-8 for all three widely used fluorescent channels: Cy3, Cy5 and FITC (Figure 1(E)). Previously, it has been shown that Au metal efficiently quenches the emission of many fluorophores [11] and conjugated polymer fluorescence, such as pyrene [19], poly(p-phenyleneethynylene) [20], cyclodextrin [21], poly(9,9*'*-bis(6-N,N,N-trimethylammonium)-hexyl)-fluorene phenylene [22], *etc.* In this case, the drastically quenched autofluoresce can be rationalized due to photo-induced electron transfer from the SU-8 units to close proximity of AuNPs [19,21]. Furthermore, performance of AuNPs as energy acceptors is efficiently enhanced, owing to the high absorption cross section of the AuNPs [23]. In addition to the energy transfer, the effect would be related to reflectivity of AuNPs when plane waves of light impinge on a mirror surface of the deposited gold layer, leading to the rejection of light from the system. The deposition of a thin layer of AuNPs facilitates easy attachment of DNA probes onto the Au surface through the formation of self-assembled monolayers (SAMs) via thiol chemistry. The immobilization process is simple and less laborious than other reported chemical modifications [5,7,24], or UV linking methods [25].

**Figure 2:**
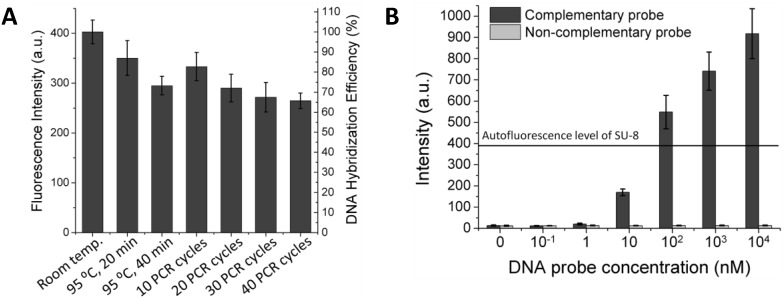
(**A**) Investigation of thermal stability of the immobilized DNA probe. After treatment at different temperatures, the immobilized DNA probe was hybridized with its Cy5-tagged complementary 45-mer sequence and scanned for the fluorescence intensity. (**B**) Sensitivity of a DNA hybridization assay on the Au-coated SU-8 surface.

After depositing the AuNPs layer and immobilizing the DNA probes, the DNA probe-carrying microparticles were exposed to different temperatures. Hybridization experiments were then performed by incubating the particles with the probe’s complementary sequence and non-complementary sequence (as a control) tagged with Cy5. Figure 2(A) shows that the immobilized DNA probes are thermally stable over a wide range of temperatures and that more than 65% of the DNA probe molecules remain on the surface without losing their biological function even after 40 repeated heating/cooling PCR cycles. With the suppressed fluorescence, the 45-mer oligonucleotide can be visualized distinctly at 1 nM concentration after hybridization to its complementary DNA probe immobilized on the AuNPs-coated SU-8. This improves the fluorescence detection limit by about two orders of magnitude compared to the untreated SU-8 (Figure 2(B)), or about 50-times compared to DNA hybridization results using SU-8 as a substrate as reported previously [5]. Furthermore, fluorescence signals obtained for the control experiment indicate that non-specific binding is negligible. 

**Scheme 2:**
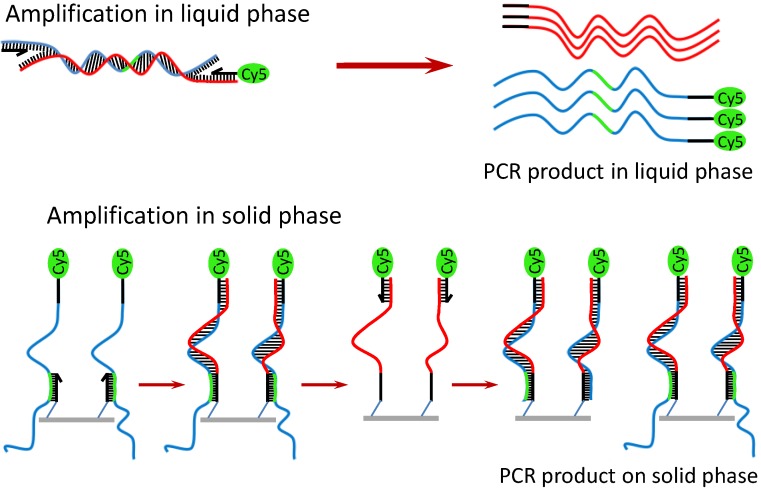
Working principle of SP-PCR using entire genomic DNA.

**Figure 3:**
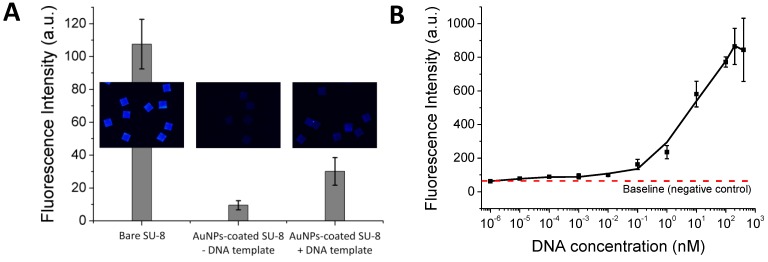
SP-PCR is performed with 2 ng/µL genomic DNA purified from *C. jejuni* (**A**), and different molar concentrations of a pre-amplified 302-bp DNA fragment of the 16S Ribosomal gene (**B**). NC: negative control.

Owing to the benefit of the thermally stable DNA immobilization, SP-PCR could be performed on the Au-coated polymer surface [26]. A schematic description and the working principle of the SP-PCR are presented in Scheme 2. In principle, the target DNA is first amplified with PCR primers (one primer is labeled with Cy5) in the liquid phase. Simultaneously on the solid phase, the amplified PCR amplicons interact with the nested DNA probes immobilized on the AuNPs/SU-8 substrate. If the immobilized probes match the sequence of the DNA templates, they are extended by the polymerase, and in turn, the extended probes serve as a template for the second strand elongation primed by the liquid phase primer, thus generating new templates for the SP-PCR. After the reaction, PCR products labeled with Cy5 remain attached to the substrate and can be visualized directly by the fluorescence scanner. The method offers several advantages over conventional multiplex PCR: less competition between different primer pairs, thus increasing multiplexing capability; only a single wavelength optical readout is needed for the multiplexing detection; and it is less time-consuming, owing to a reduction of the post-PCR gel electrophoresis. Therefore, the SP-PCR will be useful for development of point-of-care devices for sensitive detection and identification of genetic materials. In this study, two SP-PCR experiments were carried out for the detection of *C. jejuni*. In the first experiment, the SP-PCR was performed with 2 ng/µL genomic DNA of *C. jejuni*, of which the complete sequence is 1,641,481 bp in length [27]. As presented in the above section, it should be noted that the primers were used with a different molar ratio. This is because the enzymatic amplification in liquid phase is thermodynamically more favored than in the solid phase. An equal or excessive molar concentration of the liquid-phase primers can therefore inhibit the solid-phase amplification. Figure 3(A) shows that the SP-PCR product can be discriminated from the control sample. It shows that without the reduced autofluorescence, the SP-PCR signal could not be measured. However, the positive SP-PCR signal is weak. This may be either because the 302-bp DNA copies were not amplified well enough in the liquid phase to generate a clear PCR signal on the solid phase or because the amplified 302-bp DNA fragment is unable to bind with the immobilized probe for the enzymatic elongation due to its size, surface diffusion or steric hindrance effect. To examine this issue, a pre-amplified 302-bp DNA fragment was used instead of the entire genomic DNA in the second SP-PCR experiment. In this case, the forward primer is not necessary for solid-phase amplification if the immobilized probes match the DNA templates. Figure 3(B) shows a dose response curve generated by plotting the average fluorescence intensity against the log of the DNA concentrations, ranging from 0 to 400 nM. The plot clearly indicates that the 302-bp DNA fragment bound with the immobilized probe and served as the template for the probe elongation by the polymerase in the solid phase, giving a wide dynamic range, spanning from lowest detectable concentration of 0.01 nM to 200 nM. Although SP-PCR was successfully performed for the small DNA target of 302 bp, further improvements are necessary if entire genomic DNA sequences or clinical samples are used. For example, the correct primer ratio and thermal cycling program should be optimized; immobilized DNA probes with oligo (ethylene glycol) or a spacer-linker approach could be used to improve accessibility, as well as reduce steric hindrance with their template [28]. Further applications of the encoded SU-8 microparticles can be simultaneous analysis of multiple proteins, gene expression analysis, drug discovery assays, *etc.* Moreover, each encoded microparticle can have a different DNA probe attached, thus giving it an even greater multiplexing capability for the genomic studies using DNA hybridization and SP-PCR assays. 

## 4. Conclusions

We have shown that deposition of a thin AuNPs layer onto the SU-8 surface significantly reduces the autofluorescence of the SU-8 surface and improves sensitivity of fluorescence-based detections. DNA probes could be stably immobilized onto the AuNPs-coated SU-8, facilitating sensitive bioanalytical applications, such as DNA hybridization and SP-PCR. As a result, a 45-mer oligonucleotide could be detected distinctly at 1 nM concentration by the simple DNA hybridization assay. Furthermore, we also showed for the first time that a 302-bp DNA fragment could be identified by the SP-PCR on the AuNPs-coated SU-8 substrate with the sensitivity down to sub-picomolar level. We anticipate that the deposited AuNPs layer could potentially play a role of transducing agents, *e.g*., for localized surface plasmonic resonance or surface-enhanced Raman spectroscopies, within the polymer microdevices. The approach is simple and easy to perform, making it suitable for various Bio-MEMS and LOC devices that use SU-8 as a structural material.
